# Sorption of Heavy Metals (Pb, Cd, Co, and Zn) by Bacteria of the Genus *Bacillus*: An Investigation of the Ability and Consequences of Bioaccumulation

**DOI:** 10.1155/ijm/4067880

**Published:** 2025-09-18

**Authors:** Sergey A. Peshkov, Hike N. Nikiyan, Aleksey N. Sizentsov, Olga K. Davydova

**Affiliations:** ^1^Department of Chemistry, Orenburg State University, Orenburg, Russia; ^2^Department of Biophysics and Condensed Matter Physics, Orenburg State University, Orenburg, Russia; ^3^Department of Biochemistry and Microbiology, Orenburg State University, Orenburg, Russia

**Keywords:** atomic force microscopy, FTIR spectroscopy, heavy metals, morphology of bacteria, probiotics

## Abstract

The concentrations of heavy metal salts (Pb, Cd, Co, and Zn), which have an inhibitory effect on the growth of *Bacillus* bacteria, were determined using the method of successive dilutions. The dynamics of changes in the pH of the nutrient medium during bacterial cultivation with metal salts were analyzed. Bacterial growth phases were identified before and after exposure to minimally suppressive concentrations of these heavy metals. Atomic adsorption analysis was used to measure the amount of Zn, Co, Cd, and Pb accumulated by the bacteria from the medium. Both the supernatant and the biomass were analyzed at the onset of the stationary phase of microorganism growth. The effect of these elements on the morphology of bacteria was investigated using atomic force microscopy, and FTIR spectroscopy revealed differences in the shape of metals accumulated by bacteria.

## 1. Introduction

Industrial development and increased extraction and processing of metals have led to an increase in their amounts in soil and contamination [1–3]. This is especially concerning with regard to heavy metals, which are involved in the biosphere cycle and pose a major environmental challenge. Most metals accumulate in the upper humus horizons and are removed very slowly during plant consumption, leaching, erosion, and deflation of soils [3, 4]. Metals can enter the body of animals and humans through food chains, but only a small amount can enter through the lungs [5, 6]. Exposure to heavy metals leads to such adverse effects such as weight loss and decreased immunity [7–9].

The main source of heavy metals entering the atmosphere is anthropogenic [10]. The anthropogenic share of cobalt in the environment is approximately 10%, nickel 30%, cadmium and mercury 50%, copper and zinc 75%. The largest share of emissions into the atmosphere is typical for lead. According to various sources, it ranges from 70% to 90% [11, 12]. The main anthropogenic sources of heavy metal pollution are oil refineries and mining companies [13], chemical industries, metal factories [14], and transport. Metals do not decompose in natural environments and, unlike organic substances, are only redistributed. Some metals enter endorheic reservoirs, where they accumulate and, entering into chemical reactions, form toxic compounds that can serve as sources of secondary pollution of the environment. Metals are poorly removed from the soil by erosion, consumption by plants, and leaching [15]. The half-life of heavy metals depends on the initial concentration contained in the soil and can be average: for zinc, up to 270 years; for lead, up to 3300 years; and copper, up to 900 years [16, 17].

There is a significant amount of experimental data available on bacteria, plants, fungi, and their waste products, which can accumulate heavy metals [18–20]. The use of probiotic bacteria (probiotics) is particularly promising as they act as bioaccumulators for heavy metals [21, 22]. These bacteria exhibit a strong antagonistic activity against pathogenic and opportunistic microorganisms, as well as high enzymatic activity and immunostimulating effects. In addition to these characteristics, these probiotic strains also exhibit an antitoxic effect by actively accumulating and eliminating heavy metals from the body [18, 23, 24].

In this regard, studying the ability of microorganisms in probiotic preparations to accumulate heavy metals is an important task for evaluating the effectiveness of their use in treating heavy metal poisoning. Another significant issue is determining the mechanism of metal salt action on microorganisms [8, 25, 26]. In the future, this could serve as a basis for developing improved probiotic medications for treating and preventing intestinal infections while also removing toxic substances, such as heavy metals, from the body [27, 28].

The aim of this work was to determine the sorption characteristics of bacteria, specifically *Bacillus* bacteria (*Bacillus subtilis* 7048, *Bacillus licheniformis* 7038, and *Bacillus clausii* 11117), with respect to heavy metals (Zn, Co, Cd, and Pb) in vitro. The choice of these metals was based on their toxicity to cells, with lead and cadmium being selected due to their high toxicity [29, 30] and zinc and cobalt being selected as more commonly occurring biogenic elements [31].

## 2. Materials and Methods

### 2.1. Microorganisms and Metal Salts

Three probiotic strains of the *Bacillus* genus were used in the experiment: *B. clausii* 11117, *B. subtilis* 7048, and *B. licheniformis* 738. Water-soluble metal salts, including zinc sulfate, lead nitrate, cobalt sulfate, and cadmium sulfate, were used as regulatory factors in the experiment.

### 2.2. Assessment of Metal Tolerance and Impact on Microbial Growth

The minimum nontoxic concentration (MNC) of each metal salt for bacterial growth was determined through the use of the sequential dilution method. A nutrient medium containing a known concentration of each metal salt (20 mM) was prepared, and a series of double dilutions was made from this medium, after which the strains were inoculated. The absence of bacterial growth in the test tubes was considered the MNC.

The effect of heavy metal salts on bacterial growth was assessed by measuring the optical density of a bacterial suspension using a spectrophotometer (UNICO 2800, United States) at 600 nm, with an optical path length of 10 mm. The optical density is directly proportional to the concentration of cells in the medium. To do this, a specific amount of the metal of interest was added to the bacterial culture medium, and the optical density was measured every 3 h until a stationary phase was reached. A stationary phase is defined as three or more consecutive readings with the same optical density value.

Quantitative determination of the concentration of metals absorbed by bacteria was performed using an atomic absorption spectrophotometer (AASP) KVANT-2AT (Russia). Both the biomass and the supernatant were analyzed. One of the metals analyzed was added to the nutrient medium, and the microorganisms were cultured until the stationary growth phase was reached. Samples were then processed according to a standard procedure and analyzed using the atomic absorption spectrometer [32, 33].

### 2.3. Preparation for Atomic Force Microscopy (AFM)

The influence of metals on the morphology of bacterial cells was investigated using AFM. Bacteria were grown in liquid culture medium containing MNC metal salt for 36 h, and then the cells were washed to remove the nutrient medium and remaining metal salts. A 1 mL microbial suspension was centrifuged at 10,000 RPM for 5 min, and the supernatant was collected and diluted to its original volume with distilled water. This process was repeated three to four times. Finally, a drop of the washed sample was applied to fresh mica chips and dried. The samples were then scanned using a SMM-2000 (PROTON-MIET, Russia) contact scanning probe microscope with MSCT cantilevers from Bruker (United States) with a nominal stiffness of 0.01 N/m and probe radius of curvature 10 nm. Quantitative morphometric analysis of the obtained images was performed using standard software [34].

### 2.4. Preparing for FTIR Spectroscopy

Metals can accumulate in cells both in the form of ultrafine particles and in poorly soluble chelate forms. Significant differences in metal accumulation by bacteria were detected using Fourier-transform infrared (IR) spectroscopy with the use of the IRTracer-100 instrument (Shimadzu, Japan) and FSМ 2201/2202 (InfraSpec, Russia) in the range of 400–4000 cm^−1^. Preparation for analysis was carried out as follows: 5 mL of the suspension after culturing was centrifuged at 10,000 RPM for 5 min. Then, similar to preparation for AFM, the supernatant was removed and washed three to four times with distilled water. After that, the tubes containing the resulting suspension with a small amount of alcohol were placed in an ultrasonic bath at 60°C for 30 min. As a result of this process, sediments were caused by the destruction of cellular membranes and the transition of all contents into solution. Then, the washing was repeated one to two more times. A part of the resulting suspension was dried and tabletized in KBr.

### 2.5. Statistical Data Processing

STATISTICA software (V10.0, StatSoft Innovations; Tulsa, Oklahoma, United States) was used for statistical analysis. Statistical processing of the obtained data was carried out using standard mathematical methods (Student's *t*-test and the probability criterion *p* < 0.05 were assumed to be sufficient for significant differences between the experimental and control groups). The relationship between the features studied was analyzed using “correlation analysis” and “variance analysis” functions in the AtteStat program (Center for Statistical Technologies LLC). Correlation coefficients were calculated using Pearson's method with a confidence level of 0.01 or 0.001.

## 3. Results

### 3.1. Establishment of MNC

MNC is a main indicator that characterizes the resistance of bacteria to various concentrations of metal salts.  Table 1 shows a comparison of the obtained values of MNC for heavy metal salts in *B. subtilis* 7048, *B. licheniformis* 7038, and *B. clausii* 11117.

From the data obtained (Table 1), it follows that the species *B. clausii* 11117 is the most sensitive to the effects of heavy metal salts. It is impossible to clearly identify the most resistant bacterial species of the genus *Bacillus* to all the metals studied, since different types of bacteria grow at different concentrations of metal salt. The greatest toxicity to the studied bacteria of the genus *Bacillus*, according to the values of MNC (Table 1), is shown by cadmium, and the least is by lead.

### 3.2. Dependence of the pH of the Medium on the Time of Cultivation

The process of metal accumulation by bacteria occurs in an aquatic environment. When the pH of the water solution of certain salts containing the studied metals rises to pH ≥ 8, hydrolysis and precipitation occur, which can affect the accuracy of experimental results.

It is known that, as a result of the vital activity of bacteria, acidity can increase [35, 36]. Optimal conditions for the growth of bacteria of the genus *Bacillus* are within the pH range of 5.5–8.5 [37] and at a concentration of sodium chloride up to 7%. Thus, the optimal pH value for the growth of microorganisms is 6.8 [38, 39]. However, these bacteria can survive in both acidic (below 5.5) and alkaline (above 8.5) conditions within a range of 0.5–1 pH [40]. Such conditions can reduce microbial activity, but they are not harmful.

To avoid obtaining inaccurate results when using AFM and atomic absorption analysis, the pH of the nutrient medium was monitored during bacterial cultivation.

**Figure 1:**
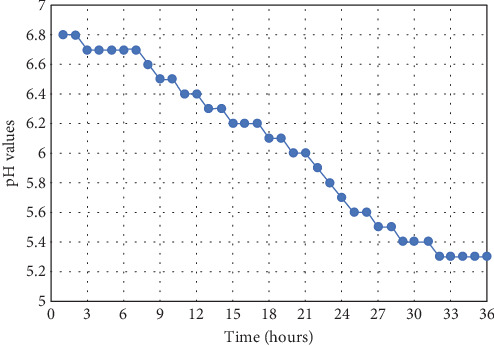
pH values of the nutrient medium from the time of bacterial cultivation.

It was found that the growth of microorganisms leads to acidification of the medium and a decrease in pH value. Thus, at 0 h of cultivation, pH was 6.8, and by the beginning of the stationary growth phase, it was 5.5 (Figure 1). The decrease in pH is not related to external conditions, particularly the dissolution of atmospheric carbon dioxide in the nutrient medium. In the blank experiment, test tubes with nutrient media without bacteria or metal salts were put in the thermostat simultaneously with test tubes containing bacteria. It was found that, in the absence of bacteria, the pH of the solution shifted slightly from an initial value of 6.8 to 6.7–6.6 within 36 h. So conditions for bioaccumulation work at pH that do not introduce additional error in metal content measurement. Based on the obtained data, pH values between 6.5 and 6.8 were used to prepare solutions of metal salts.

### 3.3. Establishment of Growth Phases

During life, microorganisms consume a nutrient substrate in which toxic metabolic products accumulate. All this leads to bacteria starting to detoxify the environment and looking for new sources of nutrients. Therefore, the accumulation of heavy metals by bacteria occurs during the stationary growth phase [41]. It is therefore relevant to determine growth phases before and after the introduction of MNC heavy metal salts to identify the optimal growth time for batch culture. The absolute values of the optical densities of the suspensions, as well as the comparisons of the effects of heavy metal salts on bacterial growth, are presented in the supporting information (Figures S1–S4 and Tables S1–S3). Analysis of growth phases (Table 2) showed that the lag phase lasts no more than 3 h for the studied strains of *Bacillus*. The exponential growth phase lasts 24 h, and the stationary phase begins after 27 h of cultivation.

Cultivation of bacteria in the presence of metals changes the optimal bacterial growth time and the onset and duration of growth phases for different *Bacillus* species. The data presented in Table 3 indicates that zinc and cobalt ions have a negative effect on the growth rate of *B. licheniformis* 7038 and inhibit the onset of the stationary phase, increasing the exponential phase from 27 to 30 h. Cadmium increases the exponential phase up to 30 h. The presence of lead does not significantly affect the phases or growth rates of all crops considered. The exponential growth phase for these strains was 27 h in the absence of cadmium. In the presence of cadmium, the time for exponential growth increased for *B. subtilis* 7048 up to 28 h and for *B. clausii* up to 36 h. In the case of zinc and cobalt, the stationary phases of *B. subtilis* 7048 and *B. clausii* 11117 began at 31–40 h. The lag phase duration for all strains did not exceed 5 h. The obtained time values are necessary for further experiments, as the determination of the amount of metal accumulated by bacteria using AASP was carried out during the stationary growth phase.

### 3.4. Accumulation of Metal by Bacteria

The next step was to determine the amount of metal accumulated by the bacteria in the nutrient medium we studied. We had previously conducted a study on the initial nutrient substrate to determine if it contained any of the metals we were interested in. Using AASP analysis, we found that the substrate did not contain any of the listed metals.

According to the data obtained using AASP, all the studied microorganisms actively accumulate lead ions from all analyzed metals, up to 53% of the introduced lead salt concentration in the nutrient medium (Table 4). Based on these results, we can create a series of bacteria, including *B. subtilis* 7048, *B. licheniformis* 7038, and *B. clausii* 11117. In this series, the amount of metal extracted from the medium decreases from left to right. These bacteria also actively accumulate cobalt (17%–27% of MNC), while zinc accumulation is worse (up to 10%–15% of the metal salt introduced). Cadmium accumulation is insignificant (2%–7% of MNC).

Analysis of the data on dynamic growth indicators (Table 5) for the studied microorganisms in the presence of heavy metals reveals a significant increase in optical density at the plateau phase. We hypothesize that this phenomenon is not due to pronounced growth stimulation, but rather to metal sorption, which causes changes in optical density for both individual bacterial cells and the entire population.

Based on the data obtained, it can be concluded that only two heavy metals from the considered group are intensively extracted from the culture medium. Lead accumulates the most actively, followed by cobalt. The remaining two metals (zinc and cadmium) accumulate insignificantly due to their low MNC values.

*B. subtilis* 7048 is the best biosorbent for lead salts (53% of MNC), while *B. licheniformis* 7038 is best for cobalt salts (27% of MNC).

Summarizing the results, we can conclude that lead accumulates in cells more than other metals (Table 4). This may be due to the formation of very stable compounds with lead, as it is a heavy metal with a large amount in the environment [42, 43]. Cells try to neutralize lead as quickly as possible by binding it to metabolic products, including amino acids. Other metals may accumulate in a similar manner. Another way for lead and other metals to bioaccumulate is through indiscriminate binding to negatively charged compounds such as phospholipids in bacterial cell membranes [44, 45]. Zinc also deserves attention because it is part of the group of biogenic elements, but its presence in *B. subtilis* 7038 and *B. clausii* 11117 has a surprising effect, contrary to what should be expected.

### 3.5. The Influence of Metal Salts on the Morphology of Bacterial Cells

The effect of heavy metal salts on the morphology of *Bacillus* bacteria was studied using AFM. *B. licheniformis* strain 7038 was chosen for analysis. The metal concentrations were selected based on MNC data obtained.

**Figure 2:**
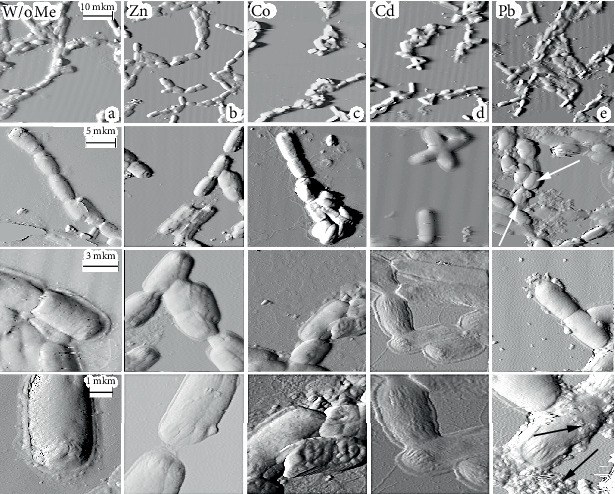
Images of *B. licheniformis* 7038 bacteria obtained using atomic force microscopy: (a) intact cells and (b–e) cells cultured in a medium containing metal salts. White arrows indicate structures with nontypical morphology, and black arrows show the formation of ultrafine metal complexes.

A series of images of experimental and control samples were obtained, with a total number of at least 30 analyzed microorganisms in each group. The results are shown in Figure 2. The concentrations of all studied metals correspond to minimal suppressed values, and there are no significant changes in bacterial cell walls. This is confirmed by the AFM images. Almost all bacteria retain their surface structures in the presence of metal salts, including pili (or villi) covering the cell surface.

The formations found on the surfaces of experimental samples marked with arrows in Figure 2e appear to be metal compounds, salts, or complexes of organic substances and metal ions. Their sizes vary depending on the type of metal and range from 80 to 180 nm. At the same time, all metals except lead do not selectively bind to bacteria, and the number of particles on the substrate is approximately equal to that on the cell surface. As can be seen in Figure 2e, bacteria incubated with lead salts actively bind to the metal, with only a small amount detected on the substrate. It is noteworthy for samples containing cadmium and lead that there are “stretch marks” at the ends of bacteria cells. Additionally, structures similar to bacteria cells were found in the presence of cadmium salts, although they have unusual sizes (see Figure 2e). Presumably, these structures are spores, which form under unfavorable environmental conditions [46, 47]. The number of spores in experimental samples can be up to 5% of total microbial cells, and they have a more rounded shape.

Additionally, it is worth noting the quantitative changes in the experimental group of microorganisms.  Table 6 provides morphometric parameters for bacterial cells of the *B. lichenformis* 703 strain after 36 h of culture with metal salts.

In comparison with the control group, there are changes in the size of microorganisms in all experimental groups. Moreover, the length of bacterial cells decreases more significantly in the presence of metal salts compared to their width, and there is a greater variation in cell size relative to the average in the presence of these salts compared to the control. This is because some bacterial cells try to adapt to adverse environmental conditions by reducing their contact area with their surroundings in order to reduce the negative impact. The most significant effect on cells is observed in the presence of lead salts, although there are quantitative differences in morphological parameters between the groups. However, cells from the experimental group do not undergo any qualitative changes to their bacterial surface structure.

### 3.6. Identification of Differences in the Shape of Metals Accumulated by Microorganisms

Previously, IR spectra of amino acids with metals have been studied using quantum chemical calculations. It is important to note that the key frequencies *ν*(NH_2_) = 3424–3324 cm^−1^, *ν*(C = O) = 1697–1708 cm^−1^, *δ*(NH_2_) = 1575–1589 cm^−1^, *ν*(NC − CO) = 1338–1317 cm^−1^, and *δ*(H − C − H) = 1384–1357 cm^−1^ when calculating vibrational spectra for various amino acids (glycine, leucine, valine, and serine) in the MP2/6-31G(p,d) approximation with metal cations do not depend on the presence of the metal and are approximately the same (Δ*ν* = 10 cm^−1^) for all amino acids. It can be expected that a similar trend will be observed for other amino acids and their metal complexes, regardless of the nature of the metal.

In the theoretical calculation of the IR spectra of bimolecular complexes, there are weak combined frequencies with the greatest contribution from the Me-N and Me-O vibrations in the range of 400–600 cm^−1^, combined with the NH_2_, CH_3_, and CH_2_ torsional oscillations. In the range of 600–700 cm^−1^, there is only one torsionally vibrating frequency at *ν*(NH_2_) = 636 cm^−1^, corresponding to the NH_2_ functional group with a nitrogen atom bonded to a metal atom. The other frequencies in the 600–700 cm^−1^ region are not torsional.

It can be assumed that in the experimental IR spectra of the degraded biomass after cultivation with metal salts, a peak at a frequency of 657–669 cm^−1^ can be attributed to the torsional oscillation of the NH_2_ group. The intensity of this torsional vibration can be used to determine the strength of the bond between nitrogen and metal atoms. Peaks in the range of 400–600 and 800 cm^−1^ are associated with valence and deformation vibrations of bonds such as Me-N and Me-O, as well as *δ*(Me-O-R) and *δ*(MeNH) bonds. These peaks are also present in other substances with high concentrations of hydroxyl groups.

**Figure 3:**
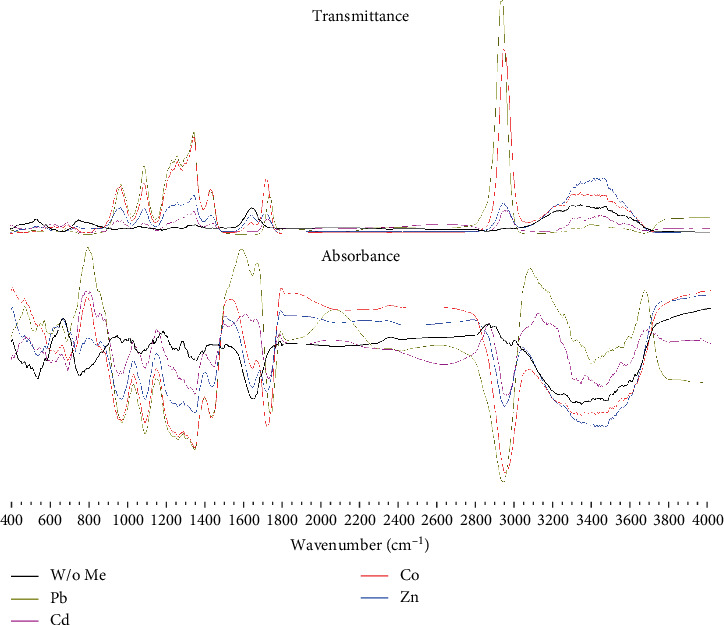
Differences in the infrared (IR) spectra of bacterial biomass after it was destroyed after culturing with various metal salts.

The most significant differences in the spectra are seen in two regions: frequencies between 500 and 1600 cm^−1^ corresponding to the combined vibrations of different functional groups in the amino acid carbon skeleton (*ν*[NC-CO], *δ*[N-C-CO], and *δ*[O-C=O]) and high-frequency vibrations between 2800 and 3800 cm^−1^ (*ν*[NH_2_] and *ν*[C-H]) (Figure 3).

This study demonstrates the accumulation of metals inside bacteria and shows that metals can accumulate in various forms. Presumably, the chelated form is formed first as it is a forced reaction that occurs through an ion exchange mechanism. Metals inevitably bind to polar residues of amino acid molecules, phospholipids, teichoic and lipoteichoic acids, and other biologically active molecules within the cell. A second form of metal is the oxide, but due to the presence of oxygen in the environment, it cannot exist in its metallic form. Therefore, the most likely form is Me-O, which is formed through redox reactions involving enzymes. This proves the possibility of using bacteria for green synthesis of nanoparticles, although the yield is lower compared to the amount of metal salts consumed.

## 4. Discussion

The analysis of experimental data aimed at assessing the sorption capacity of the studied microorganisms for tested heavy metals indicates a pronounced accumulation of lead by all strains studied in the range from 43.9% to 53.2% of the total volume introduced into the nutrient substrate. In absolute terms, the maximum sorption rates were recorded in the strain *B. subtilis*, significantly *p* < 0.001, exceeding similar indicators by 79.39% for *B. clausii* and by 75.93% for *B. licheniformis*.

As part of a comprehensive analysis of the patterns of accumulation of heavy metals using AFM and AASP, the following order of metal accumulation by microbial cells was established: Pb > Co > Zn > Cd. We assume that the observed pattern is due to a combination of several factors: physicochemical factors determining the strength of interaction between heavy metal ions and biomolecules [48] and biological factors taking into account the effect of heavy metals on vital cell functions [49, 50].

When analyzing the physicochemical patterns of bioaccumulation of heavy metals, it is necessary to proceed from several assumptions about the mechanisms of accumulation of metals by microorganisms. First, metal cations selectively attach to charged phosphoric acid residues of phospholipid molecules, teichoic (lipoteichoic) acid, and proteins [51, 52]. Such metal complexes accumulate both on the surface and inside the cell wall of the bacterium in a nontoxic form for the microorganism. With this attachment, the permeability of membranes may be disrupted, and its mobility may decrease. Accumulation of metals on the cell surface adversely affects microorganisms. This causes bacteria to adapt to new conditions, leading to a decrease in cell size and possibly a change in the structure of the cell membrane. Some bacteria turn into dormant forms—spores. The second is the penetration of metal cations into cells, where they selectively bind to organic ligands (amino acids and nucleic acids), leading to damage to DNA [53] and disruption of enzymes. Bacteria must adapt to new conditions to survive, such as increasing the number of proteins and peptides [54]. Third, bacteria can bind heavy metal ions using specific mechanisms [55], including special low-molecular weight proteins with a high cysteine content—metallothionein [56] or siderophore [57].

The importance of the stability factor of a complex should be noted, but it cannot be the only factor determining the effectiveness of metal bioaccumulation [58, 59]. In all cases, similar patterns of metal ion binding were observed: Zn^2^^+^ ≤ Co^2+^ < Pb^2+^ (the results for Cd^2+^ deserve a separate analysis). The energy preference for a complex is largely determined by the thermodynamic stability of a metal ion in an aqueous medium. The more stable the aqua complex, the more energy is needed to break the hydrate shell, which affects the energy of the metal–biomolecule complex [60–62]. For example, Ca^2+^ > Sc^2+^ > Ti^2+^≈Mn^2+^> V^2+^ > Cr^2+^ > Fe^2+^ > Co^2+^≈Zn^2+^ > Ni^2+^ > Cu^2+^ [63, 64]. For elements with higher atomic numbers, this effect is even more pronounced. It is interesting to note that this series agrees well with the spectrochemical series of metals described in other papers [65, 66].

In addition, it is known that heavy metals are toxic in their cationic form. The formation of complexes is a reversible process. Therefore, metal ions bound to biomolecules must be converted into an inactive, safe form for bacteria. Apparently, one important mechanism of cell protection is adsorption of metal complexes on the surface of cell membranes [67, 68].

Experiments using AFM confirmed the hypothesis about the accumulation of metals on the surface of bacteria, but did not show what form they existed in. Based on experimental data obtained, it can be assumed that metal compounds Co, Zn, Pb, and Cd accumulate on the surface *of B. licheniformis* 7038 (Figure 2) bacteria. Images obtained using AFM show a large accumulation of lead, which agrees with the results of spectrophotometric analysis using ASFP. There is also a significant amount of material on the cell surface associated with lead compounds, as found by other authors [67, 68].

**Figure 4:**
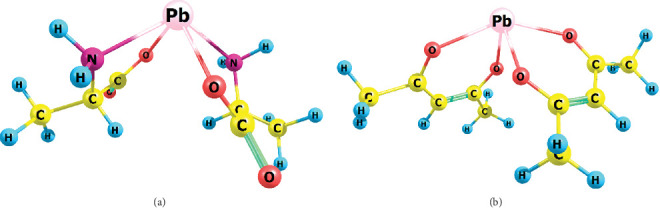
Pb(II) complexes optimized in the B3LYP/DZP approximation with L-alanine: (a) PbAla_2_ and (b) acetylacetone Pb(acac)_2_.

In this paper, we have not investigated the ability of metal ion complexes with biomolecules to form a new phase. Obviously, this scientific task is important and relevant, and the development of our research in this direction is very promising. So far, we can only assume that the good aggregation capacity of lead complexes may be related to their structural features. As indicated above, the coordination environment of the lead ion differs significantly from that of other ions (Figure 4). In lead complexes with a pyramidal structure, the central atom has many possibilities for lead–lead or lead–organic ligand interactions of another complex, that is, such a structure is favorable for intermolecular interactions and, consequently, for the growth of a new phase on the surface of the cell membrane.

It is interesting to note that the toxicity of metals to living organisms increases in the order Zn > Co > Pb > Cd [69, 70]. This series does not distinguish between eukaryotes and prokaryotes, but it correlates well with patterns of changes in energy levels of metal ion complexes and hydration energies of metal ions. Lead ions are highly toxic as cations, so the more toxic a metal, the quicker it must be neutralized. If a metal ion becomes bound, for example, by binding to metabolic products, it becomes harmless to bacteria [71]. If the resulting compounds aggregate well, for example, on the surface of the cell membrane, then the mechanism of intoxication becomes especially effective. In our opinion, a favorable combination of two physicochemical factors, the high strength of the complex and its ability to form aggregates, determines the overall high efficiency of lead bioaccumulation by bacteria.

Complexes of zinc and cobalt ions should have similar formation energies, but the slightly higher bioaccumulation efficiency of cobalt may be related to aggregation. Firstly, Co has a coordination number of 6, whereas Zn has a number of 4. This means that Co has more possibilities for intermolecular association [72, 73]. In addition, cobalt has three unpaired electrons, while zinc has none, so its high-spin state is favorable for intermolecular binding [74]. Although this is just an assumption, it deserves further research.

The low efficiency of cadmium accumulation (as a percentage of MNC) can be explained by taking into account the extreme toxicity of salts of this metal to bacteria of the genus *Bacillus*. Cadmium toxicity can be compared to the bactericidal effect of silver, so cadmium inhibits bacterial growth in even small amounts [75, 76]. The example of cadmium shows the importance of biological factors in bioaccumulation. Cadmium can form stronger complexes with molecules that make up cells, such as phospholipids and amino acids [48], affecting the mobility of cytoplasmic membranes and transport of substances across the cell membrane. The addition of the cadmium cation to a protein leads to a change in its working conformation, and the high stability of the formed complexes with cadmium makes these processes irreversible. As a result, the protein becomes inactive and the transport of substances across the membrane is inhibited.

In conclusion, it should be noted that significant differences in absolute values primarily depend on the concentration of metals in the substrate and their level of resistance (Table 1). The level of sorption of the remaining metals tested by the studied microorganisms was Cd < Zn < Co < Pb in ascending order.

Correlation analysis revealed a pronounced relationship between the level of metal concentration introduced into the substrate and the level of sorption capacity in all studied strains (*p* < 0.01). The *B. clausii* strain also revealed a direct correlation between optical density, metal concentration in the substrate (*p* < 0.05), and metal sorption (*p* < 0.01), which is consistent with our hypothesis about the influence of metals on dynamic growth indicators based on measurements of optical density of populations.

## 5. Conclusions

As part of the implementation of tasks set for us in vitro model experiments, it was found that all studied strains of *Bacillus* sp. have sorption potential with respect to tested heavy metals. This can potentially be used as a means of detoxifying ecological systems at different levels of organization.

Determination of the tolerance level of the studied strains to heavy metals has allowed us to determine the toxicity level of these elements and the threshold values of working concentrations for metal salts in order to implement subsequent stages of research. It was experimentally found that essential (Zn and Co) and conditionally essential (Cd) elements have pronounced inhibitory effects on the studied strains. We believe that this phenomenon is due to a lack of effective detoxification mechanisms for these elements. Xenobiotic elements (Pb) have no bactericidal effects in concentrations ranging from 50·10^−4^ to 200·10^−6^ mol/L, and their toxicity levels for the strains are Pb > Co > Zn > Cd, with the highest tolerance recorded in strain *B.subtilis* 7048 and the lowest resistance in *B.clausii* 11117.

The conducted study is aimed at assessing the sorption capacity of the studied *Bacillus* sp. strains. It allowed us to establish that all bacterial populations tested under conditions of a massive cationic load on the substrate actively sorbed lead from 43.9% (*B. clausii* 11117) to 53.2% (*B subtilis* 7048). This is an absolute value of 0.726 and 3.523 g/L, respectively. Such a significant difference in values is due to the amount of element introduced into the substrate. Correlation analysis shows a direct relationship between the dose of element and the level of sorptive capacity (*p* < 0.01). The order of accumulation of elements from highest to lowest corresponds to their resistance data: Pb > Co > Zn > Cd.

The analysis allows us to state, with a high degree of confidence, the fact of detoxification mechanisms in the studied strains of *Bacillus* sp., in relation to xenobiotic elements, based on a lead research model. The described data are in good agreement with the results of studying cell morphology using AFM. Qualitative and quantitative changes in morphological characteristics of bacteria cultured in the presence of metal salts are shown. At the same time, lead accumulates more actively on the surface of bacteria compared to other metals, reducing the length and width of cells and leading to the formation of spores.

The results obtained show the possibility of using probiotic bacterial strains to correct the body's condition in case of heavy metal poisoning. In addition, photographs obtained with a scanning probe microscope indicate the possibility of the formation of ultrafine forms of metal compounds using bacteria studied in this work, demonstrating the potential for their use in “green” synthesis processes. Thirdly, bacteria or bacterial isolates isolated from specific geological locations, such as *Bacillus*, can be utilized in bioremediation efforts.

## Figures and Tables

**Table 1:** MNC of heavy metal salts, ×10^−4^ mol/L.

**Test strains**	**Pb**	**Zn**	**Co**	**Cd**
*B. clausii* 11117	50	1.56	12.5	0.391
*B. licheniformis* 7038	50	25	12.5	0.391
*B. subtilis* 7048	200	3.13	12.5	1.56

**Table 2:** Growth phases of microorganisms before and after the introduction of MNC of heavy metal salts, hours.

	***B. subtilis* 7048**			***B. licheniformis* 7038**			***B. clausii***		
	**lag**	**exp**	**stat**	**lag**	**exp**	**stat**	**lag**	**exp**	**stat**
Control	3	24	27	3	24	27	3	24	27
Pb	3	27	30	3	24	27	3	27	30
Cd	3	30	33	3	30	33	3	33	36
Co	3	30	33	3	30	33	3	27	30
Zn	3	30	33	3	27	30	3	30	33

*Note:* “exp” indicates the duration of the growth phase, and “stat” indicates the beginning of the stationary growth phase. For “lag,” the specified value is ≤ 3.

Abbreviations: exp, exponential phase; lag, lag phase; stat, stationary phase.

**Table 3:** Influence of metals on the population density of studied microorganisms at maximum growth stage under periodic cultivation conditions.

**Metal salt**	**Microbial strain**	**Optical density of the population at stationary growth phase**		
		**OD control**	**OD experience**	**Percentage difference**
Pb	*B. clausii*	0.160 ± 0.001	0.327 ± 0.001^∗∗∗^	51.07
	*B. licheniformis*	0.160 ± 0.002	0.298 ± 0.003^∗∗∗^	46.31
	*B. subtilis*	0.161 ± 0.004	0.266 ± 0.002^∗∗∗^	39.47

Co	*B. clausii*	0.160 ± 0.001	0.297 ± 0.002^∗∗∗^	46.13
	*B. licheniformis*	0.160 ± 0.002	0.282 ± 0.003^∗∗∗^	43.26
	*B. subtilis*	0.161 ± 0.004	0.198 ± 0.003^∗∗^	18.69

Zn	*B. clausii*	0.160 ± 0.001	0.232 ± 0.003^∗∗∗^	31.04
	*B. licheniformis*	0.160 ± 0.002	0.216 ± 0.001^∗∗∗^	25.93
	*B. subtilis*	0.161 ± 0.004	0.283 ± 0.006^∗∗∗^	43.11

Cd	*B. clausii*	0.160 ± 0.001	0.259 ± 0.003^∗∗∗^	38.22
	*B. licheniformis*	0.160 ± 0.002	0.073 ± 0.001^∗∗∗^	54.38
	*B. subtilis*	0.161 ± 0.004	0.197 ± 0.005^∗∗^	18.27

⁣^∗∗^*p* < 0.01; ⁣^∗∗∗^*p* < 0.001.

**Table 4:** Content of heavy metals in the biomass of bacteria of the genus *Bacillus* after cultivation in a liquid nutrient medium.

**Metal salt**	**Microbial strain**	**Amount of metal in biomass**	
		**In % of MNC**	**Grams per liter**
Pb	*B. clausii*	43.9	0.726 ± 0.005^∗∗∗^
	*B. licheniformis*	51.2	0.848 ± 0.005^∗∗∗^
	*B. subtilis*	53.2	3.523 ± 0.025

Co	*B. clausii*	25.3	0.189 ± 0.001^∗∗∗^
	*B. licheniformis*	27.2	0.203 ± 0.001
	*B. subtilis*	17.6	0.066 ± 0.0003^∗∗∗^

Zn	*B. clausii*	10.1	0.010 ± 0.00005^∗∗∗^
	*B. licheniformis*	17.9	0.068 ± 0.0003
	*B. subtilis*	8.6	0.008 ± 0.00004^∗∗∗^

Cd	*B. clausii*	4.2	0.001 ± 0.000003^∗∗∗^
	*B. licheniformis*	—	Not reliable
	*B. subtilis*	7	0.004 ± 0.00001

*Note:*⁣^∗∗∗^*p* < 0.001, determination of reliably significant differences in relation to the maximum accumulation indicator.

**Table 5:** Correlation dependence of the main studied indicators.

	***B. subtilis* 7048**			***B. licheniformis* 7038**			***B. clausii***		
	**Metal concentration in the substrate**	**Optical density**	**Metal sorption (g/L)**	**Metal concentration in the substrate**	**Optical density**	**Metal sorption (g/L)**	**Metal concentration in the substrate**	**Optical density**	**Metal sorption (g/L)**
Metal concentration in the substrate	1.00			1.00			1.00		
Optical density at stationary growth phase	0.41	1.00		0.28	1.00		0.64^∗^	1.00	
Metal sorption (g/L)	0.99^∗∗^	0.36	1.00	0.85^∗∗^	0.29	1.00	0.90^∗∗^	0.74^∗∗^	1.00

⁣^∗^*p* < 0.05; ⁣^∗∗^*p* < 0.01.

**Table 6:** Morphological characteristics *of B. licheniformis* 7038 after 36 h of cultivation with metal salts.

	**Length, μm**	**Width, μm**	**Height, nm**
Control	3.9 ± 0.3	2.2 ± 0.1	513 ± 21
Pb	3.3 ± 0.4	1.6 ± 0.1	538 ± 93
Zn	3.3 ± 0.7	1.8 ± 0.3	653 ± 104
Co	3.1 ± 0.5	1.9 ± 0.3	581 ± 63
Cd	3.4 ± 0.4	1.9 ± 0.1	640 ± 22
Cells of unknown morphology (Pb)	2.8 ± 0.2	1.6 ± 0.1	1120 ± 170

## Data Availability

The data that support the findings of this study are available from the corresponding author upon reasonable request.

## Supporting Information

Supporting Information Additional supporting information can be found online in the Supporting Information section. Figure S1: The optimal growth time for bacteria of the genus *Bacillus*. Figure S2: The effect of heavy metal salts on the growth dynamics of *B. licheniformis* 7038. Figure S3: The effect of heavy metal salts on the growth dynamics of *B. subtilis* 7048. Figure S4: The effect of heavy metal salts on the growth dynamics of *B. clausii*. Table S1: The effect of heavy metal salts on the growth dynamics of *B. subtilis* 7048. Table S2: The effect of heavy metal salts on the growth dynamics of *B. licheniformis* 7038. Table S3: The effect of heavy metal salts on the growth dynamics of *B. clausii*.
