# Supplementary material for: Sorption of Heavy Metals (Pb, Cd, Co, and Zn) by Bacteria of the Genus Bacillus: An Investigation of the Ability and Consequences of Bioaccumulation
Source: Int J Microbiol. 2025 Sep 18;2025:4067880. doi: 10.1155/ijm/4067880 (PMC12463537; doi:10.1155/ijm/4067880)
Supplement: Supporting Information — Additional supporting information can be found online in the Supporting Information section. Figure S1: The optimal growth time for bacteria of the genus Bacillus. Figure S2: The effect of heavy metal salts on the growth dynamics of B. licheniformis 7038. Figure S3: The effect of heavy metal salts on the growth dynamics of B. subtilis 7048. Figure S4: The effect of heavy metal salts on the growth dynamics of B. clausii. Table S1: The effect of heavy metal salts on the growth dynamics of B. subtilis 7048. Table S2: The effect of heavy metal salts on the growth dynamics of B. licheniformis 7038. Table S3: The effect of heavy metal salts on the growth dynamics of B. clausii. [file 4067880.f1.docx]

**Supplementary Materials**

Figure S.1 – The optimal growth time for bacteria of the genus Bacillus

1– *B.clausii*; 2 – *B.licheniformis 7038*; 4 – *B.subtillis* 7048

Figure S.2 – The effect of heavy metal salts on the growth dynamics of *B.licheniformis* 7038

Figure S.3 – The effect of heavy metal salts on the growth dynamics of *B.subtillis* 7048

Figure S.4 – The effect of heavy metal salts on the growth dynamics of *B.clausii*

Table A.1 – The effect of heavy metal salts on the growth dynamics of *B.subtilis* 7048

| In relative units | | | | | |
| --- | --- | --- | --- | --- | --- |
| Time, hrs. | Control | Dynamics of changes in the optical density of cells in a liquid nutrient medium with heavy metal salts | | | |
|  |  | Pb(NO_3_)_2_ | CdSO_4_ | ZnSO_4_ | CoSO_4_ |
| 0 | 0 | 0 | 0 | 0 | 0 |
| 3 | (0.028±0.001) | (0.034±0.001) | (0.003±0.005) | (0.107±0.012) | (0.041±0.005) |
| 6 | (0.041±0.003) | (0.074±0.001) | (0.067±0.010)** | (0.145±0.003)*** | (0.079±0.006)** |
| 9 | (0.061±0.003) | (0.097±0.003) | (0.077±0.007) | (0.166±0.003)*** | (0.081±0.002)* |
| 12 | (0.081±0.003) | (0.143±0.003) | (0.107±0.003)** | (0.177±0.006) | (0.103±0.004)*** |
| 15 | (0.091±0.005) | (0.197±0.003) | (0.137±0.006)** | (0.187±0.005) | (0.116±0.005)*** |
| 18 | (0.113±0.003) | (0.204±0.004) | (0.154±0.005) | (0.21±0.003) | (0.128±0.003) |
| 21 | (0.138±0.006) | (0.210±0.006) | (0.171±0.003) | (0.233±0.002) | (0.141±0.007)*** |
| 24 | (0.144±0.006) | (0.218±0.006) | (0.175±0.003) | (0.249±0.003)** | (0.162±0.003)** |
| 27 | (0.147±0.003) | (0.241±0.006) | (0.179±0.003)*** | (0.265±0.003)** | (0.183±0.002)*** |
| 30 | (0.155±0.004) | (0.254±0.003) | (0.195±0.006)*** | (0.271±0.004)*** | (0.185±0.002)** |
| 33 | (0.159±0.005) | (0.265±0.001) | (0.197±0.002)*** | (0.278±0.001)*** | (0.195±0.011)*** |
| 36 | (0.16±0.003) | (0.266±0.003) | (0.198±0.009)*** | (0.279±0.002)*** | (0.196±0.001)*** |
| 39 | (0.161±0.004) | (0.266±0.002) | (0.197±0.005)*** | (0.283±0.006)*** | (0.198±0.003)** |
| *P≤ 0.05, **P≤ 0.01, ***P≤ 0.001 | | | | | |

Table A.2 – The effect of heavy metal salts on the growth dynamics of *B.licheniformis* 7038

| In relative units | | | | | |
| --- | --- | --- | --- | --- | --- |
| Time, hrs. | Control | Dynamics of changes in the optical density of cells in a liquid nutrient medium with heavy metal salts | | | |
|  |  | Pb(NO_3_)_2_ | CdSO_4_ | ZnSO_4_ | Pb(NO_3_)_2_ |
| 0 | 0 | 0 | 0 | 0 | 0 |
| 3 | (0.029±0.001) | (0.109±0.003) | (0.014±0.007) | (0.077±0.007) | (0.061±0.007) |
| 6 | (0.068±0.003) | (0.129±0.003) | (0.04±0.003) | (0.093±0.006) | (0.083±0.003) |
| 9 | (0.071±0.002) | (0.143±0.006) | (0.042±0.007)* | (0.113±0.003) | (0.115±0.002) |
| 12 | (0.084±0.003) | (0.165±0.003) | (0.047±0.006)** | (0.14±0.003) | (0.134±0.006)** |
| 15 | (0.094±0.005) | (0.187±0.003) | (0.05±0.009) | (0.157±0.003) | (0.153±0.003) |
| 18 | (0.126±0.003) | (0.195±0.003) | (0.051±0.006) | (0.175±0.003) | (0.176±0.003)** |
| 21 | (0.137±0.002) | (0.201±0.003) | (0.054±0.003)*** | (0.186±0.003) | (0.202±0.009)** |
| 24 | (0.139±0.004) | (0.241±0.003) | (0.057±0.003) | (0.197±0.003) | (0.241±0.003)** |
| 27 | (0.149±0.006) | (0.281±0.003) | (0.06±0.003)*** | (0.197±0.003) | (0.256±0.003)*** |
| 30 | (0.148±0.013) | (0.289±0.001) | (0.067±0.008)** | (0.201±0.001)*** | (0.275±0.003)*** |
| 33 | (0.154±0.001) | (0.296±0.003) | (0.069±0.003)*** | (0.212±0.003)*** | (0.281±0.003)*** |
| 36 | (0.159±0.003) | (0.297±0.006) | (0.072±0.003)** | (0.215±0.003)*** | (0.281±0.003)*** |
| 39 | (0.16±0.002) | (0.298±0.003) | (0.073±0.001)*** | (0.216±0.001)*** | (0.282±0.003)*** |
| *P≤ 0.05, **P≤ 0.01, ***P≤ 0.001 | | | | | |

Table A.3 – The effect of heavy metal salts on the growth dynamics of *B.clausii*

| In relative units | | | | | |
| --- | --- | --- | --- | --- | --- |
| Time, hrs. | Control | Dynamics of changes in the optical density of cells in a liquid nutrient medium with heavy metal salts | | | |
|  |  | Pb(NO_3_)_2_ | CdSO_4_ | ZnSO_4_ | Pb(NO_3_)_2_ |
| 0 | 0 | 0 | 0 | 0 | 0 |
| 3 | (0.029±0.007) | (0.048±0.003) | (0.127±0.005) | (0.056±0.001) | (0.105±0.006) |
| 6 | (0.068±0.006) | (0.053±0.003) | (0.149±0.013)** | (0.057±0.010)* | (0.139±0.006)** |
| 9 | (0.07±0.009) | (0.099±0.007) | (0.168±0.009) | (0.083±0.006) | (0.171±0.003)* |
| 12 | (0.084±0.007) | (0.137±0.003)* | (0.183±0.003)** | (0.144±0.003) | (0.174±0.004)*** |
| 15 | (0.094±0.003) | (0.175±0.003) | (0.208±0.006)** | (0.163±0.003)** | (0.191±0.006)*** |
| 18 | (0.121±0.003) | (0.237±0.011) | (0.21±0.006) | (0.193±0.001)*** | (0.227±0.003) |
| 21 | (0.126±0.003) | (0.249±0.003) | (0.219±0.003) | (0.195±0.006) | (0.249±0.009)*** |
| 24 | (0.137±0.006) | (0.281±0.003) | (0.224±0.006) | (0.197±0.003)*** | (0.261±0.003)** |
| 27 | (0.14±0.007) | (0.314±0.006) | (0.233±0.003)*** | (0.207±0.003)*** | (0.266±0.001)*** |
| 30 | (0.148±0.003) | (0.319±0.006)** | (0.244±0.003)*** | (0.223±0.002)*** | (0.290±0.003)** |
| 33 | (0.15±0.003) | (0.323±0.006) | (0.256±0.001)*** | (0.225±0.001)*** | (0.296±0.008)*** |
| 36 | (0.159±0.003) | (0.326±0.003) | (0.258±0.005)*** | (0.231±0.003)*** | (0.297±0.001)*** |
| 39 | (0.16±0.001) | (0.327±0.001)** | (0.259±0.003)*** | (0.232±0.003)** | (0.297±0.002)** |
| *P≤ 0.05, **P≤ 0.01, ***P≤ 0.001 | | | | | |
